# Tessier 7 Cleft: Clinical Presentation and Surgical Correction in a Case Report

**DOI:** 10.7759/cureus.71062

**Published:** 2024-10-08

**Authors:** Anup R Mannali, Pasupathy M, Satish Kumar

**Affiliations:** 1 Plastic and Reconstructive Surgery, SRM Medical College and Research Centre, Chennai, IND; 2 Cleft Lip and Palate Reconstructive Surgery, SRM Medical college Hospital and Research Centre, Chennai, IND; 3 Plastic and Reconstructive Surgery, SRM Medical College Hospital and Research Centre, Chennai, IND

**Keywords:** aesthetic, facial symmetry, oral competancy, post-operative scar, tessier cleft 7

## Abstract

Tessier 7 cleft, or transverse facial cleft, is a rare congenital anomaly involving lateral facial tissues, often resulting from embryonic disruptions in the first and second branchial arches. It presents varying severities from macrostomia to complete clefts affecting soft tissue and skeletal structures. Surgical management is challenging, requiring a multidisciplinary approach for functional and aesthetic reconstruction. This case report discusses a patient's presentation, surgical intervention, and outcomes. Our case report describes a rare form of Tessier cleft in a one-and-a-half-year-old male child with no syndromic association. This was diagnosed by clinical evaluation, and radiological modalities and managed surgically. Tessier 7 cleft, or transverse facial cleft, is a rare anomaly extending from the mouth to the ear, resulting from abnormal fusion of facial processes during embryonic development. It ranges from mild macrostomia to severe deformities affecting soft tissues and bone. Surgical management aims to restore facial symmetry, function, and aesthetics, often requiring multiple procedures. Challenges include maintaining oral competence and achieving satisfactory cosmetic results. A multidisciplinary approach is essential, involving plastic surgeons, orthodontists, and speech therapists, with long-term follow-up to monitor development and outcomes. Early diagnosis is the key to achieving an acceptable functional and aesthetic outcome in children. Surgical correction should be done at the earliest for oral competency and normal speech development.

## Introduction

Tessier 7 cleft, also known as lateral facial cleft, is an uncommon craniofacial anomaly characterized by a lateral extension of the oral commissure. It has an incidence of 1/80 000-1/300 000 live births or 0.3-1.0% of the cleft spectrum [1]. This condition manifests as facial asymmetry and exhibits varying degrees of severity, potentially affecting soft tissues, muscles, and skeletal structures. It can be seen as an isolated event, in combination with other craniofacial anomalies, or as part of syndromes such as Goldenhar syndrome/Oculo-Auriculo-Vertebral Spectrum (OAVS) and Treacher-Collins syndrome [2]. Paul Tessier introduced the classification of craniofacial clefts in 1976, outlining 15 specific types identified by Tessier numbers 0 to 14 [3]. The management of Tessier 7 cleft necessitates a comprehensive approach, encompassing meticulous preoperative evaluation, surgical intervention, and postoperative care. This case report presents a patient diagnosed with Tessier 7 cleft, delineating the clinical presentation and surgical correction. The report aims to contribute to the existing literature on this uncommon condition by providing detailed insights into the patient's history, diagnostic approach, surgical planning, and outcomes. By disseminating our experience, we endeavor to enhance the understanding of Tessier 7 cleft and its management among healthcare professionals. The surgical correction of Tessier 7 cleft encompasses various techniques, including straight-line closure, Z-plasty, vermilion square flap, and tissue rearrangement. Critical considerations during the procedure include maintaining oral competence, preserving facial nerve function, achieving facial symmetry, aligning muscles, and minimizing scarring. Postoperative management focuses on monitoring for potential complications, implementing speech therapy, and conducting regular follow-ups to assess long-term outcomes. This report will elucidate the preoperative evaluation, surgical technique employed, postoperative course, and outcomes observed in our patient. 

## Case presentation

**Figure 1:**
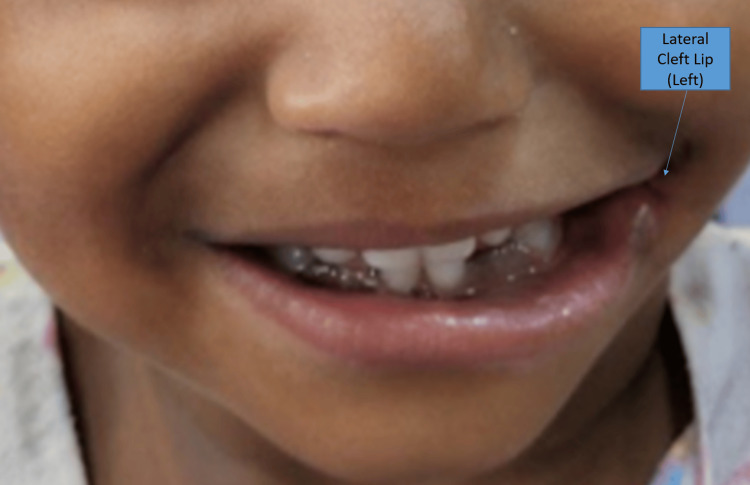
Preoperative picture of the patient with left lateral cleft lip.

One and half year old male child from Tamil Nadu, India born via full-term vaginal delivery with no perinatal complications presented with complaints of the wide opening of the mouth to the left side and an additional large fold of skin tag in front of the left side ear since birth with no other associated complaints or other abnormalities detected. There was no history of neonatal intensive care admissions. The child’s parents had a third-degree consanguineous marriage. He has one sister who is 8 months old with no anomalies. The child mostly consumes liquid food since chewing food expels most of it out of the lateral cleft lip defect. Immunization was noted to be age-appropriate. The Child has achieved appropriate milestones for the age except for delayed speech and language development. On examination of the face, there was asymmetry due to the evident discontinuity of the corner of the mouth with the vermillion of the upper and lower lip not meeting at the corner of the mouth (Figure 1). Examination of the nose, alveolus, and palate was normal. There was a preauricular skin tag with a deformed tragus on the left side.

**Figure 2:**
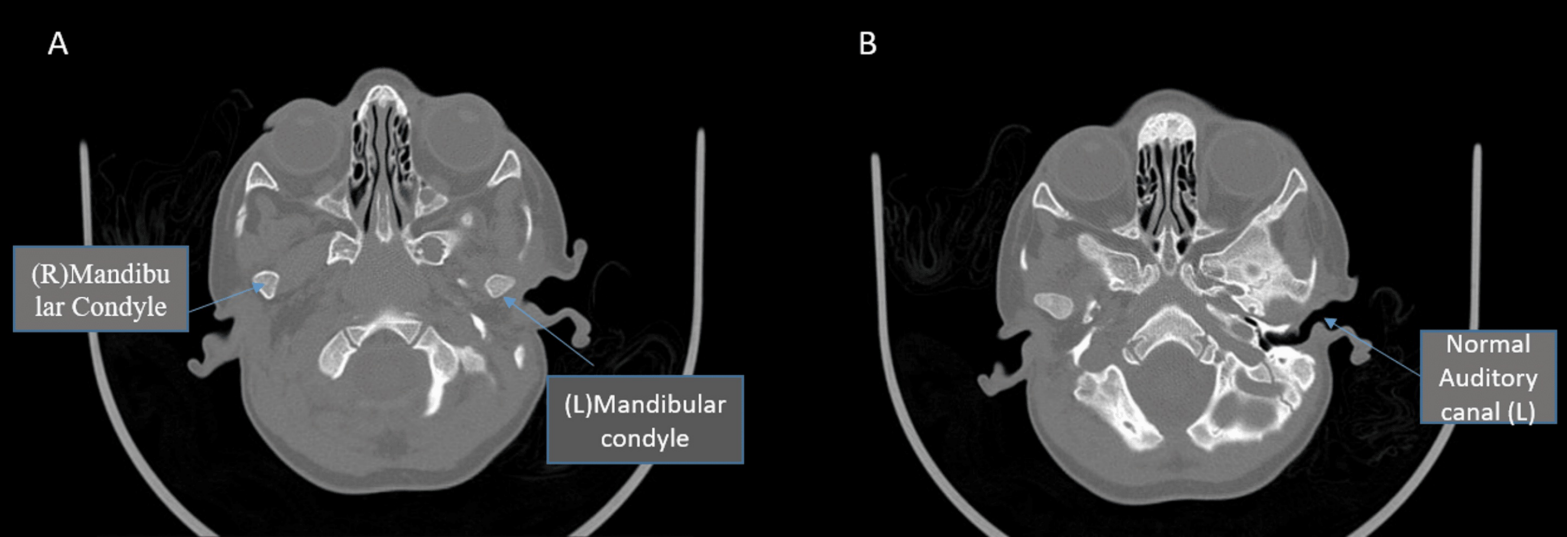
CT scan of facial bones axial view. (A) well formed right and left mandibular condyles and (B) normal auditory meatus over the left side.

CT scan revealed no bony abnormalities. The axial view showed normal mandibular condyles and normal left-side external auditory meatus (Figure 2). Based on clinical examination a diagnosis of Tessier 7 facial cleft (lateral cleft lip) was established.

USG abdomen was done at birth which was normal. 2D-echo done at birth revealed a small patent Foramen Ovale (left to right Shunt) and repeat 2D-Echo at 1 year of age revealed normal study. Syndromic associations such as Goldenhar syndrome, Dandy-Walker syndrome, Treacher-Collins syndrome, and posterior fossa malformations, hemangioma, arterial anomalies, cardiac anomalies, and eye abnormalities (PHACE) syndromes have been ruled out before taking the patient for surgical correction.

Intervention

**Figure 3:**
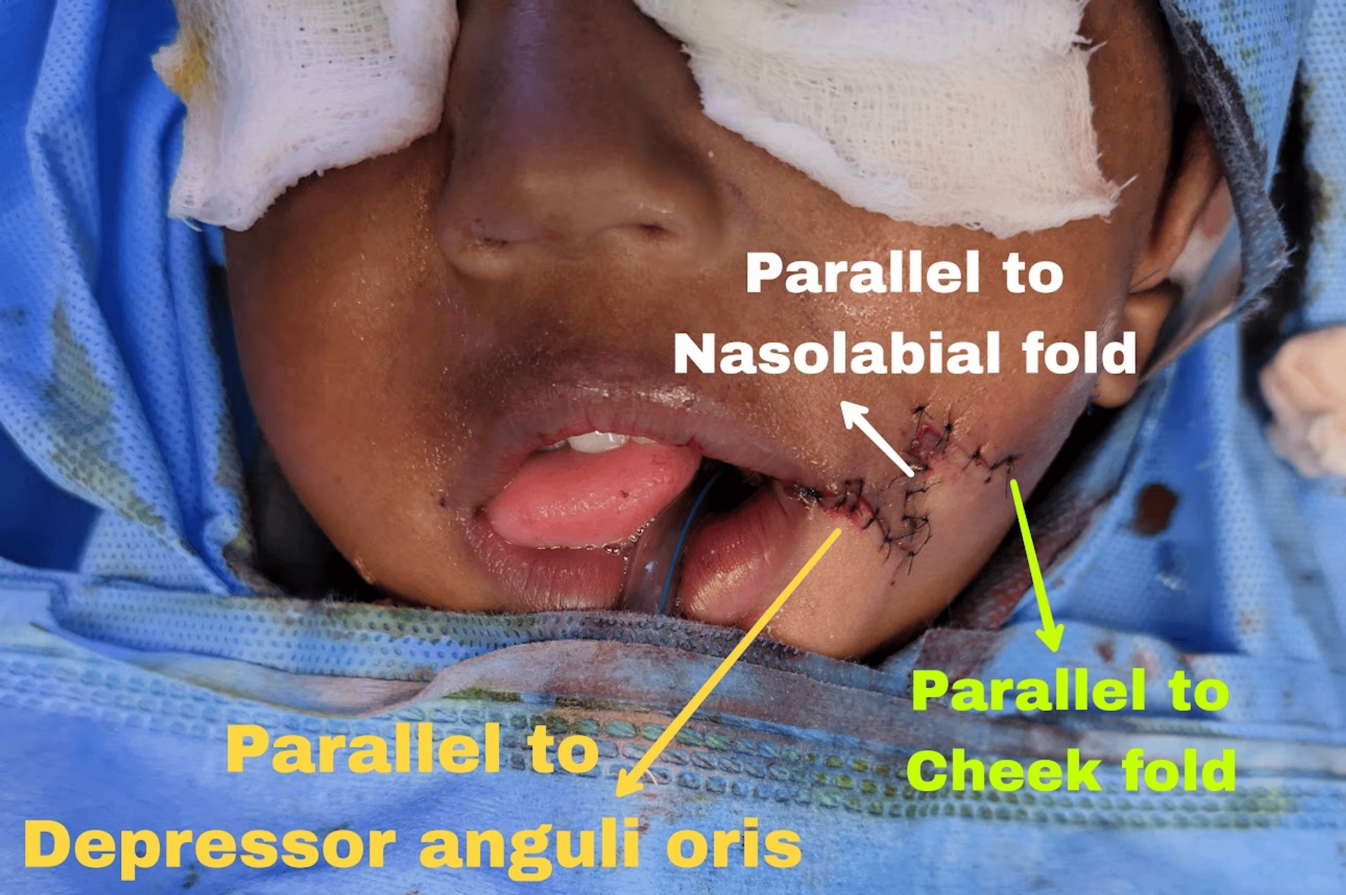
Intraoperative Picture of the patient with lateral lip cleft.

Under general anesthesia. Mucosal turnover incision made. Sutured using 4-0 monocryl. Orbicularis oris of the upper lip overlapped and sutured to the lower lip orbicularis oris to reconstruct the modiolus using 5-0 prolene. Z-plasty was done at the skin and sutured using 6-0 ethilon. Left Pre-auricular skin tag with deformed cartilage was excised and primary suturing was done (Figure 3). The patient was reversed from GA and upon extubation was shifted to the post-operative anesthesia care unit for further care and monitoring The skin sutures were removed on the sixth post-operative day.

Follow-up

**Figure 4:**
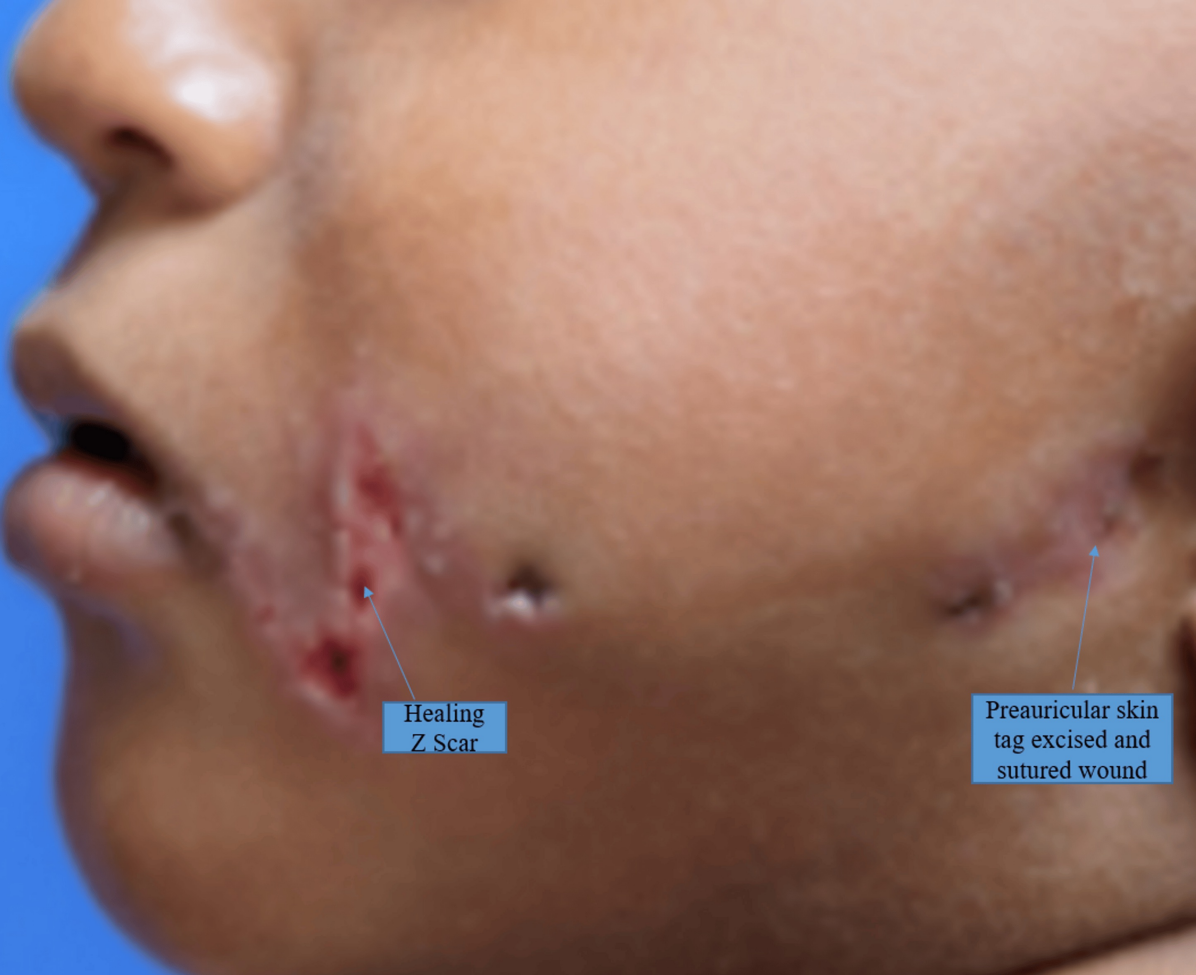
Post operative day 10. Denoting closure of the left side lateral cleft with a healing Z scar(superficial dermal loss) , well approximated upper and lower lip at the commissures and a healing preauricular skin tag excised wound.

**Figure 5:**
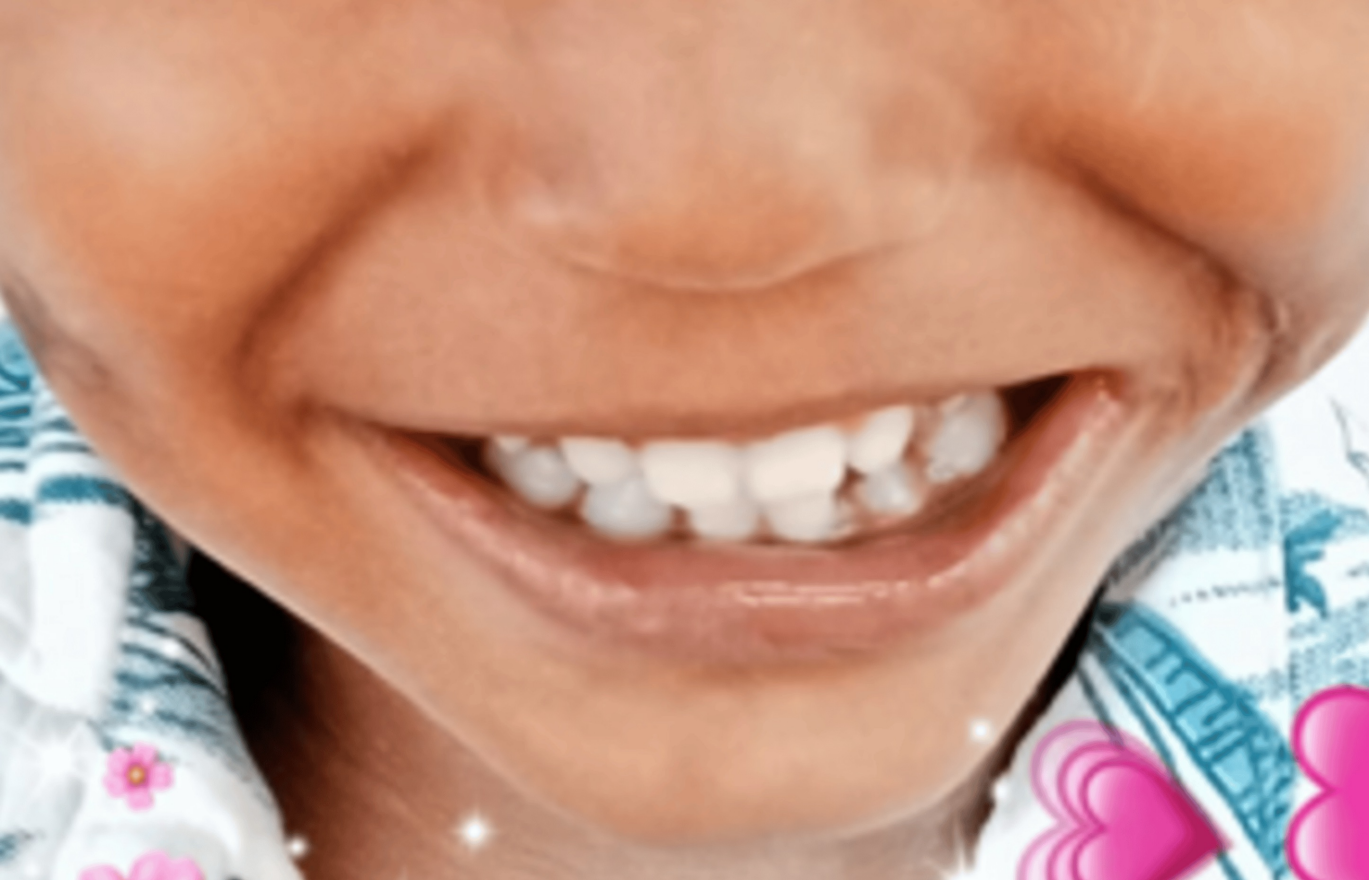
6 months postoperative picture denoting aesthetic facial symmetry and lip competancy.

The patient was discharged immediately after suture removal on the sixth post-operative day and was subsequently followed up after 10 days (Figure 4), one month, three months, and six months (Figure 5) from the day of surgery. The functional and aesthetic outcomes were achieved with respect to being able to properly chew food, attaining facial asymmetry, maintaining lip competency, etc. The surgical scar was soft and supple and the vertical limb of the Z was along the nasolabial fold thereby giving a better cosmetic outcome.

## Discussion

Macrostomia, also known as Tessier 7 cleft, is an uncommon birth defect occurring in 1/80,000-1/300,000 live births [1]. This condition arises from improper facial fusion during embryonic growth, manifesting as a fissure extending from the mouth to the ear. The severity of macrostomia ranges from mild to severe, impacting both soft tissues and bone [4]. This anomaly can appear independently or in conjunction with other facial clefts and syndromes. The majority of cases are one-sided and confined to the front edge of the masseter muscle [5]. Surgical treatment focuses on restoring symmetry, functionality, and appearance by reconstructing the orbicularis oris muscle and creating balanced commissures with minimal scarring [2].

Surgeons employ various techniques depending on the specific case and their expertise. A multidisciplinary approach involving plastic surgeons, orthodontists, and speech therapists is crucial. For achieving optimal aesthetic and functional results, early intervention and follow-up are vital thereby addressing potential complications and providing psychological support.

Our case is a unilateral left lateral lip cleft with a preauricular skin tag seen in a patient of one year and six months of age. The patient had presented with speech problems and with difficulty chewing. After the initial evaluation, syndromic associations such as Goldenhar syndrome, Dandy-Walker syndrome, and Treacher-Collins syndrome were been ruled out. CT facial bones done in this regard showed no bony abnormalities. We adopted the technique of making mucosal turnover incisions and upper and lower lip orbicularis oris sutured to reconstruct the modiolus. Z-plasty was done on the skin to prevent any straight-line contractures. The patient was followed up on the seventh day, three and six months post-surgery. We were able to attain facial symmetry, oral competence, and satisfactory aesthetic outcomes. This technique is comparable to other techniques such as vermillion flap, triangular flap, straight-line closure, and two triangular flap techniques recommended by surgeons [5,6,7,8].

The technique involving Z-plasty for skin closure is a subject of discussion among authors [9]. In our case, the aesthetic and functional outcome was acceptable in our technique of modiolus reconstruction with Z-plasty for skin closure.

## Conclusions

The critical aspect of lateral cleft lip repair is the meticulous mobilization and reconstruction of the orbicularis oris muscle, which is essential for restoring lip function and symmetry. The use of Z-plasty for closure has proven effective in achieving satisfactory cosmetic outcomes, as this technique allows for seamless integration with surrounding facial structures and minimizes scarring. This approach not only preserves the natural facial contours but also enhances long-term functional results.
